# The Use of Antibiotics as Chiral Selectors in Capillary Electrophoresis: A Review

**DOI:** 10.3390/molecules27113601

**Published:** 2022-06-03

**Authors:** Gabriel Hancu, Lajos Attila Papp, Blanka Szekely-Szentmiklosi, Hajnal Kelemen

**Affiliations:** Department of Pharmaceutical and Therapeutic Chemistry, Faculty of Pharmacy, George Emil Palade University of Medicine, Pharmacy, Science and Technology of Târgu Mureș, 540142 Târgu Mureș, Romania; gabriel.hancu@umfst.ro (G.H.); blanka.szekely-szentmiklosi@umfst.ro (B.S.-S.); hajnal.kelemen@umfst.ro (H.K.)

**Keywords:** antibiotics, chiral drugs, chiral separation, chiral selectors, capillary electrophoresis

## Abstract

Chirality is becoming an essential issue in modern pharmaceutical research as regulatory agencies emphasize the safety and efficiency of enantiomers in drug development. The development of efficient and reliable chiral separation methods became a necessity in the last 30 years, and capillary electrophoresis (CE), due to its relatively low costs and “green” features, is attracting increased attention. Cyclodextrin (CD) and their derivatives are the most frequently used chiral selectors (CSs) in CE, however, the use of antibiotics as CSs represents an interesting alternative. Various classes of antibiotics (aminoglycosides, ansamycins, glycopeptides, lincosamides, macrolides, tetracyclines) have been used more or less successfully for the enantio-separation of pharmaceuticals. Antibiotics offer the possibility of a multitude of potential interactions (electrostatic, inclusion, hydrogen bonding, etc.) due to their chemical diversity, allowing the enantio-separation of analytes with a wide range of structural characteristics. This article aims to review the application of various classes of antibiotics in the CE enantio-separation of pharmaceuticals. Antibiotic physiochemical characteristics, variables impacting enantio-separation, advantages, and disadvantages when certain antibiotics are used as CSs in CE are also explored.

## 1. Introduction

Chirality is an important issue in modern pharmaceutical research, as the desired pharmaceutical effect of a chiral drug is usually related to only one of the enantiomers, called eutomer, while the other enantiomer, called distomer is usually less active and sometimes can be responsible for the side effects observed in the case of racemate administration [1]. Pharmacological response when administering a chiral drug in the form of a racemic mixture presents inter-individual variability because enantiomers have different pharmacological and pharmacokinetic properties and the human body itself behaves like a chiral environment, being made up of a series of chiral structures: amino acids, sugars, and nucleotides. If, in an achiral environment, the enantiomers of a chiral drug have the same physical and chemical properties as in a chiral environment, such as living organisms, enantiomers can act as two distinct drugs with different pharmacokinetic and pharmacological characteristics [2].

The Food and Drug Administration (FDA) issued a set of recommendations for the pharmaceutical development of pure enantiomers and racemates in 1992. These rules stipulate that for drugs having chiral centers, absolute stereochemistry must be known, and that this information must be acquired early in the drug development process. According to the recommendations, the pharmacokinetic, pharmacological, and toxicological profiles of all the enantiomers and the racemic mixtures have to be characterized prior to drug approval [3].

Currently, there are two tendencies in the development of new chiral drugs, one involving the “chiral switch” practice in which the eutomer of an already marketed racemic mixture is introduced on the market, while the other is the development of new enantiomerically pure drugs [4]. For the development of a new enantiomerically pure drug, three strategies can be applied: to start with a pure enantiomer of a natural precursor (“chiral pool”); to use a stereoselective synthesis (including enzymatic and biological procedures); or to perform a preparative separation from the racemate obtained through a non-stereoselective synthetic protocol (“chiral resolution”) [5].

Therefore, developing new, reliable, and robust analytical methods for the chiral resolution and enantiomer determination of therapeutically active chiral drugs became a necessity and also a challenge in pharmaceutical research. Several methods for the analysis of chiral compounds are available, among these, we can distinguish high-performance liquid chromatography (HPLC), gas chromatography (GC), supercritical fluid chromatography (SFC), nuclear magnetic resonance (NMR) spectroscopy, or capillary electrophoresis (CE) [6]. The most widely used method in chiral separations is HPLC, however, the implementation of CE methods has been gaining ground in the last two decades. In the direct HPLC methods, the chiral selector (CS) is usually immobilized in a chiral stationary phase (CSP), in the case of CE the separation procedure is more straightforward by simply adding the CS into the background electrolyte (BGE) [7].

The advantages of using CE in the chiral separation of pharmaceuticals are related to the high separation efficiency, low consumption of samples, CSs, and reagents, short analysis time, ease of operation, and great flexibility in choosing and changing CSs. Additionally, we can mention CE as a “greener” alternative to HPLC due to the low consumption of organic solvents, resulting in a reduction in waste disposal [8].

A large number of CSs are currently available, and their number continues to increase; therefore, choosing the best CS for a specific purpose is a difficult issue. Among the most frequently used CS in CE we can mention cyclodextrin (CD) and their derivatives, macrocyclic antibiotics, polysaccharides, proteins, and ligand exchangers [9,10].

CDs are by far the most popular, useful, and successful CSs in CE due to their commercial availability, relatively affordable prices, low UV absorbance, stability over a wide pH range, and high capacity of complexation towards a large number of pharmaceuticals [11,12,13].

An alternative to using CDs as CSs in CE is using macrocyclic antibiotics (aminoglycosides, ansamycins, glycopeptides, macrolides, tetracyclines, and others); which can be appropriate for the enantiomeric resolution of a variety of chiral compounds, including charged (positive and negative) and uncharged analytes [14].

Several reviews have been published regarding the use of macrocyclic antibiotics as CSs in CE, among these, we can mention the ones published by Ward and Oswald [15] in 1997, Desiderio and Fanali [16] in 1998, Prokhorova et al. [17] in 2010, or Dixit and Pak [18] in 2014. We can also mention a few reviews which tackle the use of certain macrocyclic antibiotics as CSs in both HPLC and CE, among these we distinguish the ones published by Ward and Farris [19] in 2001, Aboul-Enein and Ali [20] in 2010, and Shapovalova et al. [21] in 2018. However, recent advances were made in the field of chiral separation by CE, and the reviews mentioned earlier do not characterize entirely the current “state of the art’’.

All antibiotics which are used as CSs share similar structural features, such as the presence of several stereogenic centers and many functional groups, permitting multiple interactions with the analytes. Other interactions such as ionic, hydrogen bonding, dipole–dipole, π–π, hydrophobic and steric repulsion are also important issues in explaining enantio-resolution [9].

The purpose of this article is to review the utilization of antibiotics in CE for the enantio-separation of pharmaceuticals. The practical use of enantio-separations in the case of several pharmaceuticals is explored, advantages and disadvantages, and also limitations of antibiotics used as CSs are discussed.

## 2. Antibiotics as CSs in CE

### 2.1. Glycopeptides

Glycopeptides are the most successful class of antibiotic CSs in CE. This class includes substances like avoparcin, balhimycin, eremomycin, ristocetin A, teicoplanin, and vancomycin. Glycopeptides are cyclic heptapeptides made up of an aglycon part with aminosaccharide moieties attached to it. Three or four fused macrocyclic rings (composed of linked amino acids and substituted phenols) compose the aglycon, which has a distinctive “basket” shape. The saccharide moieties connected to the aglycon “basket” can spin freely and take on a variety of shapes. They have a high number of stereogenic centers, as well as a number of functional groups that can participate in stereoselective interactions. In acidic solutions, these antibiotics absorb strongly below 250 nm and exhibit a small minimum of around 260 nm (allowing direct UV detection between 250 and 260 nm) [15,18,22].

**Figure 1:**
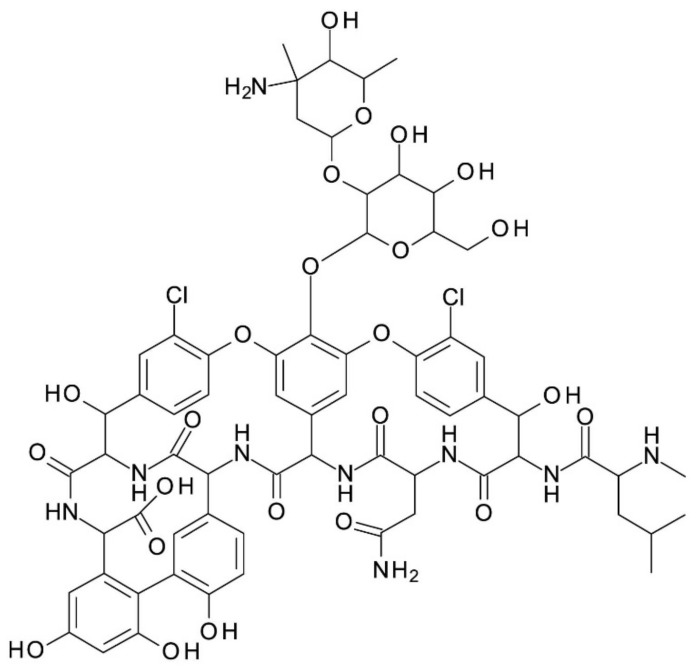
Chemical structure of Vancomycin.

***Vancomycin*** (C_66_H_75_Cl_2_N_9_O_24_) is a macrocyclic glycopeptide antibiotic produced by the soil actinobacteria *Amycolatopsis orientalis (Nocardia orientalis)*. It has an amphoteric character, its structure consisting of a seven-membered peptide chain constituted of two chlorinated beta-hydroxytyrosine molecules, three substituted phenyl-glycines, *N*-methyl-leucine, and aspartic acid amine; one of the phenyl-glycine has a disaccharide moiety composed of vancosamine and glucose attached to it. It has 18 stereogenic centers, 9 hydroxy groups, 2 amine groups (primary and secondary amine), 7 amido groups, and 2 chloride substituents on two different aromatic rings. It is used in the form of hydrochloride. Vancomycin’s chemical structure is presented in Figure 1. It has six reported pKa values: 2.9, 7.2, 8.6, 9.6, 10.5, and 11.7. Its isoelectric point (*pI*) is 7.2. It is soluble in water and polar aprotic solvents, slightly soluble in methanol and insoluble in higher alcohols. The aqueous solution of vancomycin deteriorates within 2–4 days at room temperature and 6–7 days at 4 °C [22,23].

Vancomycin is the most commonly used glycopeptide antibiotic in CE enantio-separations. Vancomycin can be employed as uncharged or charged CS based on BGE pH values, having positive or negative mobility at pH buffer values below and above 7.2, respectively, due to the presence in its structure of both amino and carboxylic groups. Electrostatic interactions are thought to have a substantial part in the mechanism of enantiomer separation, where vancomycin is used as CS, and hydrophobic and hydrogen bonding interactions are the essential secondary interactions that generate the chiral recognition process [22,23,24].

Vancomycin was the first glycopeptide employed as CS in CE; Amstrong et al. published in 1994 a study in which vancomycin was used for the chiral separation of approximately 100 chiral compounds (*N*-derivatized amino acids, nonsteroidal anti-inflammatory drugs, antineoplastic drugs, and other carboxylic acid compounds). Low concentrations of vancomycin (2–5 mM) in a 100 mM phosphate BGE over a pH range of 4–7 were used in the determinations; good resolution (in some cases above 10) but also long migration times (in some cases over 30 min) were obtained. It was assumed that the fact that vancomycin occurs as a cationic species in the utilized BGE is primarily responsible for its capacity to resolve the model anionic substances. Separations were optimized by establishing the influence of CS concentration, BGE pH, and organic modifier type and concentration (acetonitrile, methanol, 2-propanol) on the chiral resolution. The tendency of vancomycin to interact with the walls of uncoated fused silica capillaries was found to decrease separation efficiency and increase analysis times [23].

Fanali and Desiderio used vancomycin as CS for the CE enantio-separation of loxiglumide, a cholecystokinin antagonist used in the treatment of gastrointestinal pathologies. Chiral resolution was achieved in approximately 12 min using a 50 mM phosphate BGE at pH 6.0, and 3 mM vancomycin as CS. The effects of CS concentration and BGE pH on enantiomer resolution were investigated. A partial separation zone approach (polyacrylamide coating of the capillary in conjunction with “counter-flow”, while the capillary was filled with CS-containing BGE at low pressure, keeping the detector cell free of CS) was used to minimize the effect of vancomycin UV absorption, which allowed an excellent limit of detection (LOD) of 0.5 µg/mL for each enantiomer. Using the optimized conditions concentrations of 0.2% (*w/w*) distomer (*l*-loxiglumide) can be detected. The method was applied for chiral purity control in pharmaceutical preparations [25].

Vespalec et al. used vancomycin as CS for the chiral separation of amino-quinolylcarbamate derivatized amino acids containing sulfur and selenium. Separation in both uncoated and coated capillaries was investigated. The association constants of vancomycin were found to be in the same order of magnitude, as for β-CD. It was demonstrated that the chemical composition, pH, and concentration of the BGE have a significant impact on the vancomycin-analyte interactions [26]. The advantages and drawbacks of using vancomycin for the separation of amino acid derivatives were later summarized by the same research group [24].

Ward et al. used coated capillaries to suppress electroosmotic flow (EOF) and a counter-current CE separation technique to test the efficacy of vancomycin as CS. Nonsteroidal anti-inflammatory drugs and dansyl amino acids were used as model molecules. The utilized “partial separation zone technique” (“partial filling method”) improved analyte detection sensitivity significantly [27].

In the “counter-current” CE separation technique, the capillary is first filled with vancomycin-containing BGE, then negatively charged analytes are injected at the cathode of the capillary and detected at the anode; this improves detection sensitivity significantly and practically eliminates wall adsorption effects, resulting in higher resolutions and faster analysis times [28].

**Figure 2:**
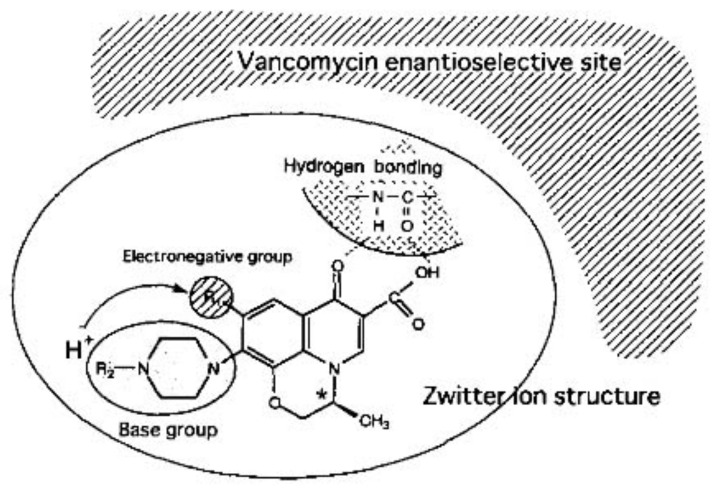
Speculative diagram depicting the possible separation mechanism between vancomycin as CS and quinolone carboxylic acids. The figure is reproduced from Arai et al. [29] with permission from Elsevier.

Arai et al. studied the chiral separation of antibacterial quinolone carboxylic acids, including ofloxacin, by CE using vancomycin as CS. The best results were obtained when using 100 mM acetate BGE at pH 4.0 and 5 mM vancomycin as CS. The molecular interaction between the quinolone carboxylic acids and vancomycin, based on structural differences, was explored, as well as the influence of experimental settings on the enantio-separation. The presumed interactions between the analyte and the CS were identified as being hydrogen bonding and amide linkages with piperidinyl, keto, and carboxyl groups; the molecular sizes of the quinolones which have to fit the hydrophobic pockets of the CS, and aromatic interaction with the naphthyl ring of the quinolones (Figure 2) [29].

Desiderio et al. used vancomycin as CS for the enantio-separation of herbicides: aryloxypropionic (mecoprop, fenoprop, and dichlorprop), *N*-benzoyl-*N*-(3-chloro-4-fluorophenyl)-2-aminopropionic acid (flamprop) and aryloxyphenoxypropionic (haloxyfop, fluazifop, diclofop, and fenoxaprop) derivatives. Because of the vancomycin positive charge and the absence/reduction in EOF at the operational pH, the detector path was kept free of absorbing vancomycin during detection by applying a partial filling technique, which produced a remarkable enhancement in detection sensitivity. CS concertation, BGE pH, and capillary temperature were found to strongly influence chiral separation. Baseline resolution was obtained for all analytes in less than 10 min using 75 mM Britton–Robinson BGE at pH 5.0 containing 6 mM vancomycin as CS. The method was used in environmental analysis to detect a soil extract treated with haloxyfop ethoxyethyl ester, which hydrolyzed to the acidic metabolite haloxyfop [30].

Fanali et al. employed the use of the partial filling technique together with capillary electrophoresis–electrospray ionization-mass spectrometry (CE–ESI-MS), for the enantio-separation of arylpropionic acids anti-inflammatory drugs (carprofen, etodolac, flurbiprofen, ibuprofen, ketoprofen, naproxen) using vancomycin as CS. At acidic pH, vancomycin electrophoretic mobility as a positively charged CS allowed avoiding contaminating the ESI-MS source with the CS. The method was used to determine enantiomers of ibuprofen and its phase I metabolites, as well as etodolac and its metabolites, in urine samples [31].

Bednar et al. evaluated the use of teicoplanin and vancomycin as CS for the chiral separation of UV nonabsorbing compounds, aspartic and glutamic acid enantiomers. A polyacrylamide-coated capillary using the partial filling-counter current method and an aqueous-organic BGE in the pH range of 4.5–6.5 composed of sorbic acid/histidine was used in the determination. The influence of CS concentration, BGE pH, organic modifier concentration, type, and concentration of absorbing co-ion (indirect UV detection) on the enantio-resolution was studied. The better results were obtained when using vancomycin (10 mM sorbic acid/histidine, pH 5.0, 10 mM vancomycin), which allowed reasonably good chiral resolution of the investigated molecules. The optimized method was applied for the analysis of real-life samples, such as teeth dentine and beer [32].

Fanali et al. studied the enantio-separation of derivatized *N*-acetyl amino acids (*N*-acetyl glutamic acid, cystine, proline, serine, tyrosine) using CE, vancomycin as CS and applying a partial-filling countercurrent technique. The effect of CS concentration, BGE pH, capillary temperature, and organic modifier on the chiral resolution was studied. Baseline separation of all five analytes was obtained using a 20 mM ammonium acetate at pH 5.0 and 2.5 mM vancomycin as CS [33].

**Figure 3:**
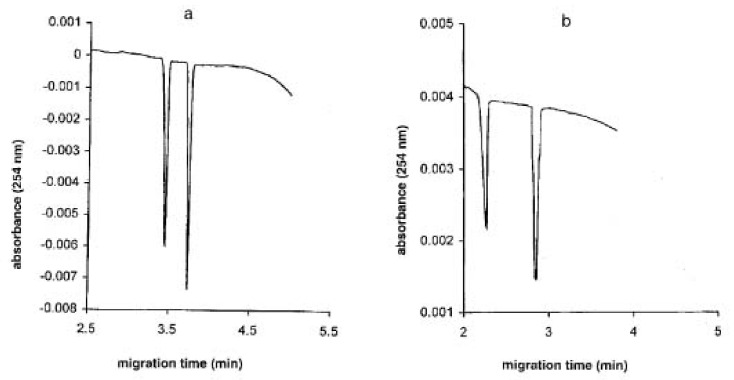
Enantiomeric separation of carboxymethylcysteine and *N*-acetamido-carboxymethylcysteine (**a**) using 10 mM vancomycin as CS; (**b**) using 1 mM vancomycin as CS. The figure is reproduced from Fanali et al. [32] with permission from Wiley.

Fanali et al. developed a CE chiral separation method for carboxymethylcysteine and *N*-acetamido-carboxymethylcysteine using vancomycin as CS. Separations were carried out in a polyacrylamide-coated capillary filled with a sorbic acid/histidine BGE at a pH range 4.5–6.5 using partial filling-countercurrent technique. In order to boost the sensitivity of the method, indirect UV detection was adopted due to the low absorption of the investigated enantiomers. The influence of CS concentration, BGE pH, organic modifier type, and concentration on enantio-resolution and migration times was investigated. Figure 3 shows the enantio-separation of the two analytes at the optimum CS concentration [34].

**Figure 4:**
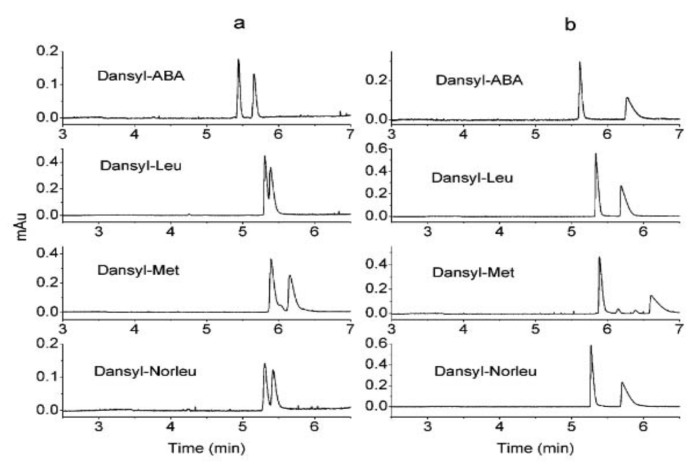
Comparison of the enantio-selectivity of balhimycin (**b**); and vancomycin (**a**) used as CS for the enantio-separation of dansylated α-amino acids (CS concentration 2 mM). The figure is reproduced from Kang et al. [35] with permission from ACS Publications.

Kang et al. published a mechanistic study of chiral separation with vancomycin and balhimycin as CS. CE was used to study the role of the sugar moiety of the two glycopeptides (vancomycin and balhimycin have the same aglycon and almost similar sugar moieties) in chiral recognition. Balhimycin’s enantio-selectivity for dansylated α-amino acids was found to be 2.6 times higher than that of vancomycin (Figure 4). When the sugar amino group of balhimycin was blocked by an *N*-carbamoylation process, the enantio-selectivity was dramatically reduced compared to vancomycin, which remained almost the same after carbamoylation. Because the dimerization characteristics of glycopeptides are connected to their glycosylation patterns, a dimerization-based mechanism was proposed to explain this phenomenon. A synergistic effect between dimerization and the formation of the glycopeptide-analyte complex can explain the correlation between high dimerization constants and improved enantio-selectivity; consequently, the chiral recognition of glycopeptides with high dimerization constants may be aided by their dimer structure. [35].

Wang et al. developed a CE method for the determination of stereoisomeric impurity of folinic acid diastereomers with vancomycin as CS. The effects of BGE concentration, BGE pH, CS concentration, organic modifier concentration, capillary temperature, and applied voltage on the enantio-resolution were studied. The best results were obtained when using 100 mM Tris-phosphate buffer at pH 6.0 containing 1 mM vancomycin and 5% acetonitrile on a poly(dimethylacrylamide) dynamically coated capillary. Folinic acid diastereomers were baseline separated in 7.5 min using the optimized conditions [36].

Zhang et al. used vancomycin as CS for the CE enantio-separation of five nonsteroidal anti-inflammatory arylpropionic derivatives (carprofen, ibuprofen, ketoprofen, naproxen, pranoprofen). A synergistic system using chiral ionic liquids (ILs) was created to decrease adsorption to the capillary wall and UV absorption of the CS, potentially lowering the quantity of vancomycin required for enantio-separation. The synergistic impact of two ILs based on amino acid ester, *l*-alanine, and *l*-valine tert-butyl ester bis (trifluoromethane) sulfonamide, with the antibiotic CS, was evaluated. The influence of several parameters, such as BGE pH and composition, CS concentration, chiral ILs concentration, organic modifier type, and concentration on the enantio-resolution was evaluated by means of Statistical Product and Service Solutions (IBM, Armonk, NY, USA). For all model analytes, the combination resulted in a considerable improvement in enantio-separation when compared with chiral Ils or vancomycin alone. The introduction of an organic solvent (methanol) helped to increase the enantio-resolution even more. The method was applied to verify the enantiomeric purity of (*S*)-naproxen in pharmaceutical preparations [37].

An application of chiral CE-ESI-MS using vancomycin as CS for the enantio-separation of amino acids was published by Sanchez-Hernandez et al. Amino acids were derivatized using 9-fluorenylmethoxycarbonyl chloride to facilitate their interaction with vancomycin and the production of precursor ions with higher m/z, which were used in MS investigations. To avoid ionization suppression and generate a suitable MS signal, the authors employed a positively charged coated capillary (polybrene-coated capillary) in combination with the partial filling technique to avoid the contamination of the ion source by the nonvolatile CS. The simultaneous enantiomeric separation of 17 amino acids (two of which were nonprotein amino acids) took around 20 min under optimal conditions [38].

***Ristocetin A*** (C_94_H_108_N_8_O_44_) is a macrocyclic glycopeptide antibiotic, produced by the bacteria *Amycolatopsis lurida (Nocardia lurida)*. The aglycon consists of four fused macrocyclic rings (12 membered, 14 membered, and two 16 membered rings), to which several sugars (arabinose, glucose, mannose, rhamnose) are covalently bonded. The four macrocyclic rings form a “basket” shape structure. It has 38 stereogenic centers, 7 aromatic rings, 6 amide groups, 21 hydroxy groups, 2 primary amino groups, and 1 methyl ester. It is soluble in acidic aqueous solutions and less soluble at neutral pH; is soluble in polar organic and insoluble in non-polar organic solvents [18,22].

Armstrong et al. were the first to test ristocetin A as CS in CE in 1995 just as in the case of vancomycin a year before. Over 120 chiral analytes were tested, including a variety of *N*-blocked amino acids, non-steroidal anti-inflammatory drugs and other compounds containing carboxylic acid groups. The influence of CS concentration, BGE pH, and organic modifier concentration on chiral resolution and migration time was studied. Because of the low amounts of ristocetin A required for enantio-resolution and its low absorbance (by comparison with vancomycin) at wavelengths higher than 250 nm, direct UV detection was possible. Enantioselective association appears to be aided by electrostatic interactions between the chiral analyte and the CS [39].

Mohr et al. studied the chiral separation of six α-hydroxy acids (mandelic acid, 4-bromomandelic acid, 3-hydroxymandelic acid, 3-hydroxy-4-metoxymandelic acid, 4-hydroxymandelic acid, and 4-methoxymandelic acid,) by capillary electrochromatography (CEC) using ristocein A as CSP (a 25 % particle loaded ristocetin A continuous bed). Negatively charged chemicals moved towards the anode under initial conditions (0.05% triethylamine and 0.1% acetic acid in methanol, normal polarity), and were carried on by the weak cathodic EOF, resulting in long analysis times of over one hour. To reverse the EOF and shorten the analysis time, a cationic surfactant cetyltrimethylammoniumbromide was used as a mobile phase additive. The shortest retention times (less than 20 min) were obtained when a mobile phase with a concentration of 25 mM cetyltrimethylammoniumbromide was used [40].

***Teicoplanin*** is a mixture of five major macrocyclic glycopeptide antibiotic analogs (Teicoplanin A_2_-1—Teicoplanin A_2_-5) and four minor ones (Teicoplanin R_S_-1—Teicoplanin Rs-4), produced by bacteria *Actinoplanes teichomyceticus*. The aglycon consists of four fused macrocyclic rings, to which three carbohydrate moieties consisting of *N*-acetylglucosmanine and mannose are attached. The length and shape of a sidechain linked to a third carbohydrate moiety *d*-glucosamine, are the sole differences between major and minor components. Teicoplanin is distinguished by the presence of an *N*-acyl hydrocarbon chain in one of its glucosamine moieties. Four of the seven aromatic rings are phenolic moieties that can be ionized, and two include chlorine substituents. Teicoplanin is considered to be a lipoglycopeptide [18,22].

MDL 63,246 (Hepta-tyr), a semisynthetic glycopeptide antibiotic structurally related to the teicoplanin family, has been tested in CE chiral separations by Fanali et al. Separations were carried out using a polyacrylamide-coated capillary in partial filling-counter current mode and aqueous-organic buffers in the pH range 4–6. The influence of CS concentration, BGE pH, organic modifier concentration, and capillary temperature on the enantio-resolution was studied. The best results were obtained at pH 5.0 using low concentrations of antibiotic (0.55–1.11 mM). The addition of an organic solvent (acetonitrile) to the BGE was necessary due to the limited solubility of Hepta-tyr in aqueous solution. Even in coated capillaries, remarkable adsorption of Hepta-tyr on the capillary wall has been reported [41].

***Avoparcin*** is a mixture of two macrocyclic glycopeptide antibiotics α-avoparcin (C_89_H_102_ClN_9_O_36_) and β-avoparcin (C_89_H_101_Cl_2_N_9_O_36_), which differ by the presence of an additional chlorine atom in β-avoparcin. It has 32 stereogenic centers, 7 aromatic rings, 6 amide groups, 16 hydroxyl groups, 3 amine groups (2 primary amines, 1 secondary amine), a carboxylic group, and 4 carbohydrate side chains [42].

Avoparcin capacity as CS in CE was tested by Ekborg-Ott et al. using as chiral model molecules *N*-blocked amino acids and aryl propionic anti-inflammatory drugs. A comparative study on the chiral separation efficiency of avoparcin, ristocetin A, teicoplanin, and vancomycin on *N*-3,5-dinitrobenzoyl-derivatized amino acids has been made; the use of vancomycin resulted in the longest migration times, ristocetin A the shortest, while the migration times obtained when using avoparcin were intermediate. No connections have been established between the efficiency/inefficiency of a certain CS towards an analyte; however, in all studied situations at least one of the tested CSs offered good enantio-resolution, while the others in the same analytical conditions offered poor separation. A low concentration of CS (0.4 mM) was used to allow direct UV detection at 254 nm. The tendency of avoparcin to bind to the capillary wall was observed [42]

***Balhymicin*** is a structural analog of vancomycin isolated from the fermentation broth of *Amycolatopsis balhimycina (Amycolatopsis mediterranei)*. Balhimycin has the same aglycon as vancomycin, made up of a heptapeptide backbone cyclized in the side chains of aromatic amino acids; the type and linking position of the sugar moieties are different. Vancomycin has a disaccharide connected to the aglycon, whereas balhimycin has two sugar moieties, a *d*-glucose and an oxo-vancosamine, coupled at two distinct locations to the aglycon. It has 17 stereogenic centers and 3 atropisomeric macrocyclic rings with a dehydrovancosamine sugar attached to it [43].

Balhimycin and its halogenate analogue bromobalhimycin were evaluated as CSs in CE by Jiang et al. A combined approach of the dynamic surface coating technique, the co-EOF electrophoresis technique, and the partial filling technique was used for the chiral separation of 16 acidic model drugs (dansyl amino acids, aryl propionic anti-inflammatory drugs). A comparative study was made on enantio-recognition capacity of balhimycin, bromobalhimycin, and vancomycin. For most of the tested compounds, balhimycin proved to have the highest enantio-resolution capacity [44].

**Figure 5:**
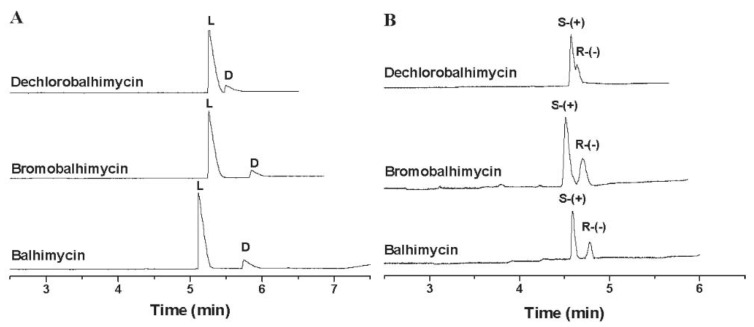
Enantio-separation of enantio-enriched *l*-dansyl-methionine (**A**); and (*S*)-ketoprofen (**B**) by CE using balhimycin, bromobalhimycin, and dechlorobalhimycin as CS (CS concentration 2 mM). The figure is reproduced from Jiang et al. [45] with permission from Wiley.

The same research group conducted a comparative study evaluating the enantio-recognition capacity of balhimycin, bromobalhimycin, and its dehalogenated analogue dechlorobalhimycin on model dansyl amino acids and aryl propionic anti-inflammatory drugs. The observed enantio-resolution capacity of balhimycin was much higher in all cases, which indicates that the chlorine substituents were important in the enantio-resolution of the model analytes (Figure 5). To explain this phenomenon, a dimerization-based mechanism was proposed; each monomer’s two chlorine substituents, which mutually penetrate the cavity of the neighboring dimer molecule, are thought to promote dimerization and, as a result, enantio-separation. The cavity generated by the aglycon is thought to be the principal site of interaction between test analytes and antibiotics, and it is this cavity that is responsible for stereoselective inclusion [45].

***Eremomycin*** (C_73_H_89_ClN_10_O_26_) is another structural analog of vancomycin, isolated from the culture filtrate of *Actinomycete* numbered INA-238. It has 22 chiral centers, 3 sugar moieties, 5 aromatic rings, a carboxylic group, 9 hydroxyl groups, 7 amido groups, and 3 amino groups [46].

Prokhorova et al. evaluated the potential of eremomycin as CS in CE using as model substances aryl propionic anti-inflammatory drugs (fenoprofen, flurbiprofen, ibuprofen, indoprofen, ketoprofen). The electrophoretic characteristics of eremomycin, as well as its stability in solution, have been studied. The effect of CS concentration, BGE type, and pH, organic modifier type and concentration on the enantio-resolution was studied. Low concentration of CS was used (2.5 mM), which allowed direct UV detection, eremomycin exhibiting low absorbance between 260 and 350 nm. The migration order of the enantiomers was established. The enantio-separations of profens in CE with eremomycin as CS added in the BGE and in HPLC with eremomycin as CSP showed comparable tendencies [47].

Prokhorova et al. also studied an interesting combination of coupled chitosan capillary coating and eremomycin CS for the CE enantio-separation of several acids (fenoprofen, flurbiprofen, ibuprofen, indoprofen, ketoprofen, mandelic acid, α-methoxyphenylacetic acid, 3-phenylbutiric acid, 2-phenoxypropionic acid). Eremomycin adsorption on the capillary wall and two types of chitosan-based coatings (chitosan and linked chitosan) were studied. The applicability of chitosan-coated capillary over fused silica capillary has been demonstrated in terms of enantio-resolution and time analysis [48].

### 2.2. Ansamycins (Rifamycins)

Ansamycins feature a unique “ansa” structure, which consists of a heterocyclic structure containing a naphthoquinone core connected by an aliphatic bridge. Because of the naphtha-hydroquinone ring, they absorb strongly in UV and generate stable yellow to red-colored solutions (depending on pH, solvents, etc.) [15,18,22].

***Rifamycin S*** and ***Rifamycin SV*** (C_37_H_47_NO_12_) are semisynthetic rifamycins obtained from **Rifamycin B,** an ansamycin produced by *Norcardia mediterranei*. By desesterification of rifamycin B and oxidation of two hydroxyl groups on the naphthalene nucleus, rifamycin S is obtained, its reduction leading to rifamycin SV. Both substances have nine stereogenic centers, two aromatic rings, four hydroxy groups, a carbohydrate moiety, two methoxy groups, an amide bond, two methoxy groups, and a carboxymethyl substituent. The structural difference between these substances is the type of the substituents on the naphtha-hydroquinone ring, which are oxy-acetic acid and hydroxyl, for Rifamycin S and Rifamycin SV, respectively. They show absorption maxima at approximately 220, 304, and 425 nm and minimum at approximately 275 and 350 nm [49].

Ward et al. evaluated the possibility of using rifamycin B and rifamycin SV as CSs in CE. Rifamycin B can exist as a divalent acid because its carboxylic and hydroxyl groups are ionizable, but rifamycin SV is preponderantly present under uncharged form at neutral and basic pH values employed in the study. When the BGE pH is decreased, rifamycin B loses a part of the negative charge on the molecule, preventing a significant charge-charge interaction with the positively charged amine-containing analytes. Rifamycin B occurs largely as a di-anion at pH 7, whereas the analyte is positively charged, resulting in a strong electrostatic interaction. Several model molecules (alprenolol, amphetamine, epinephrine, octopamine, oxprenolol, norepinephrine, normetanephrine, pindolol, propranolol) were enantio-separated using a 100 mM phosphate BGE at pH 7.0, containing 30% 2-propanol and 25 mM rifamycin B. Rifamycin B was shown to be enantio-selective for positively charged compounds, whereas rifamycin SV was found to be enantio-selective for negatively charged compounds. Rifamycin B was considered to be an ideal CS for monocyclic analytes, however bicyclic analytes, such as pindolol and propranolol, were also resolved in this study; rifamycin SV is more suitable for the separation of larger analytes containing at least two cyclic moieties in their structure (hexobarbital, glutethimide). The detection wavelength was set at 350 nm to increase sensitivity due to the decrease in baseline noise, based on a comparison with detection at 275 nm. The quantity of analyte injected into the column was kept as low as possible to improve resolution, and it has been established that indirect detection can detect as little as 0.1% of enantiomers [50].

***Rifampicin (Rifampin) ***(C_43_H_58_N_4_O_12_) has two different structural characteristics by comparison with rifamycin B: it has a hydroxyl group as a substituent, whereas the latter contains an oxy-acetic acid group, and it includes an extra methyl piperazine ring substituent. Rifampicin’s characteristic red-orange color is due to the naphthoquinone chromophore [49].

Rifampicin was evaluated as CS in CE by Dixit and Park using as chiral model basic molecules, two β-blockers (metoprolol, propranolol) and a selective serotonin reuptake inhibitor antidepressant (sertraline). The influence of CS concentration, BGE pH and composition, and applied voltage on the enantio-resolution was investigated. Baseline resolution for metoprolol and propranolol was obtained using a 100 mM phosphate BGE at pH 7.0 containing 50% 2-propanol and 20 mM rifampicin as CS; while for sertraline baseline chiral separation was obtained with 100 mM phosphate BGE at pH 7.0 containing 40% 2-propanol and 23 mM rifampicin as CS [51].

### 2.3. Macrolides

Macrolides consist of macrocyclic lactone rings containing 14, 15, or 16 atoms which allow the inclusion complexation of chiral analytes. Various deoxy-sugar or amino sugar residues are glycosidically linked to the substituted lactone macrocycle. This class includes substances, such as azithromycin, boromycin, clarithromycin, erythromycin, and gamithromycin. Macrolides interference with UV detection of analytes is lower than in the case of glycopeptides because their structure lacks aromatic rings, resulting in poorer UV absorption [15,18].

**Figure 6:**
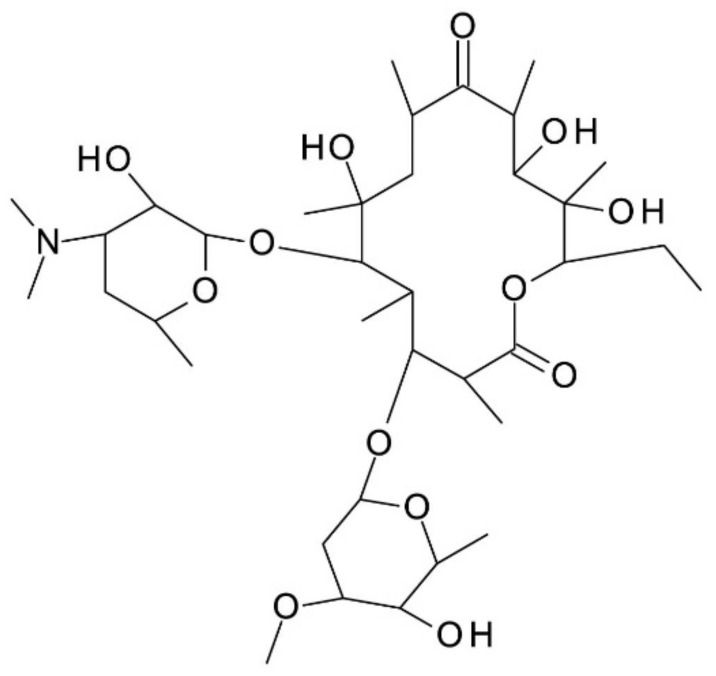
Chemical structure of Erythromycin.

***Erythromycin*** is a macrolide isolated from *Streptomyces erythreus* (*Saccharopolyspora erythraea*) containing a 14-atom macrocyclic lactone ring, with a characteristic “basket” shape. The lactonic macrocycle of erythromycin is called erythronolide, which is glycosidically bound to a 6-deoxy sugar (*l*-cladinose) and an amino sugar (desozamine). It has 10 stereogenic centers, two sugar moieties, three hydroxyl groups, a methoxy group, and a tertiary amine group. Erythromycin’s chemical structure is presented in Figure 6. It can be positively charged in acidic and neutral BGE because of its dimethyl amino group (pKa 8.8), on the desozamine moiety. However, the relative instability of erythromycin under acidic conditions represents a limiting factor of its applicability [52,53].

Ha et al. investigated erythromycin and five related substances (erythromycin *N*-oxide, anhydroerythromycin, anhydroerythromycin *N*-oxide, erythralosamine, erythralosamine *N*-oxide) as potential CS in CE. Phosphate BGEs at two different pH levels (3.0, 7.0) and sodium tetraborate BGE at pH 9.2 were used; different concentrations of CS ranging from 0.1 to 10 mM were added to the BGE. Erythromycins had stronger interactions with acidic compounds than with neutral or weakly basic chemicals, especially in an acidic medium. However, none of the 21 chiral drugs (with different structural characteristics and electrophoretic behavior) showed enantio-separation in the studied conditions; over 3000 runs were made in 70 different experimental conditions. Based on the variation in electrophoretic mobility of the compounds in the presence of different concentrations of CS, the complexation constants for the compounds that showed interaction were calculated. Computational modelling was used to determine the size of erythromycin; the aglycone ring was found to be only half the size of the β-CD cavity, which could explain why the use of erythromycin as CS had such a negative result in the investigation [53]. The lack of an organic component in the BGE and a low CS concentration may have prevented chiral separation; as the highest concentration of erythromycin in acidic and neutral aqueous medium was 10 mM, while the highest concentration in basic aqueous medium was 5 mM.

The applicability of erythromycin as CS in CE was tested by Huo et al. on four antihepatitis biphenyldimethylester derivatives. The effects of CS concentration, BGE pH, organic modifiers, capillary temperature, and applied voltage on enantio-resolution were studied. The enatio-resolution as well as migration times of the analytes, improved when CS concentration was increased. Additionally, the content of organic solvents in the BGE influenced strongly the enantio-separation. The best results were obtained when using a BGE containing 50 mM phosphate at pH 6.0 and 50% methanol, and 20 mM erythromycin as CS [54].

Erythromycin lactobionate was applied by Xu et al. as CS for the non-aqueous capillary electrophoresis (NACE) enantio-separation of six model basic chiral drugs (chloroquine, *N,N*-dimethyl-3-(2-methoxyphenoxy)-3-propylamine, duloxetine, nefopam, primaquine, propranolol). In this study, erythromycin lactobionate was used which exhibits higher solubilization in aqueous solutions, consequently, CS concentrations up to 12% were used. The influence of CS concentration, BGE pH and concentration, capillary temperature, and applied voltage on enantio-separation was tested; the results showed that CS concentration and BGE pH influenced strongly the enantio-resolution [55].

Chen et al. also employed erythromycin lactobionate as CS in NACE for the enantio-separation of two extensively used basic chiral drugs a serotonin and norepinephrine inhibitor antidepressant (duloxetine) and a β-blocker (propranolol). The best results were obtained when using a BGE composed of 50 mM Tris, 100 mM boric acid in methanol, and 100 mM erythromycin as CS. The influence of CS concentration, BGE pH and composition, organic solvent type, capillary temperature, and applied voltage on enatio-resolution and migration times was studied [56].

**Figure 7:**
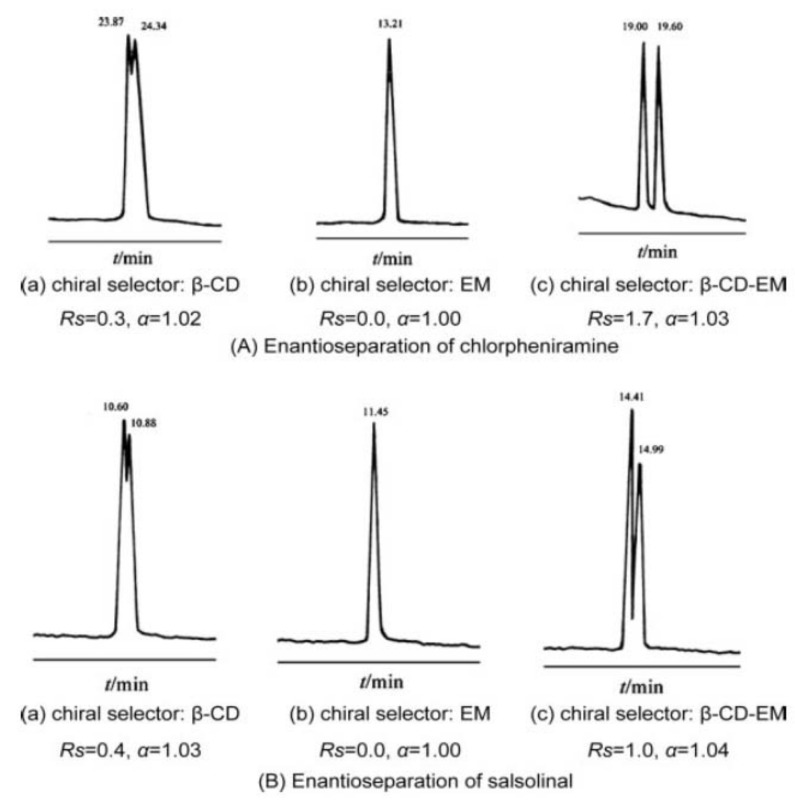
Enantio-separation of chlorpheniramine (**A**); and salsonilol (**B**) using β-CD, erythromycin (EM), and β-CD-derivatized erythromycin (β-CD-EM) (CS concentration 15 mM). The figure is reproduced from Dai et al. [57] with permission from Wiley.

An interesting idea was exploited by Dai et al., who synthetized and tested as CS a water-soluble β-CD-derivatized erythromycin. The goal of substituting 1-oxygen-2,3-epoxypropane at the main hydroxyl site of β-CD is to create a molecule that has both β-CD and erythromycin functionalities. The chemical structure of β-CD-derivatized erythromycin was confirmed using FTIR, 1H NMR, and MALDI-TOF-MS experiments. The influence of CS concentration, BGE pH and concentration, organic modifier, and applied potential, on the enantio-separation was investigated. Β-CD-derivatized erythromycin showed improved enantioselectivities compared with single β-CD and erythromycin as CS, for the enantio-separation of three model drugs (chlorpheniramine, propranolol, salsolinol) (Figure 7). The best results were obtained when using a BGE containing 20 mM phosphate at pH 3.01 (chlorpheniramine, propranolol) or pH 4.98 (salsolinol) and 20% methanol, and 15–20 mM β-CD-derivatized erythromycin [57].

***Clarithromycin*** is a semisynthetic macrolide containing a 14-membered macrocyclic lactone ring. It has two sugar moieties, four hydroxyl groups, two methoxy groups, and one tertiary amino group. Clarithromycin has various properties that make it an interesting CS candidate, such as good solubility in methanol (as a salt), low solvent viscosity, and especially low UV absorption [52].

Clarithromycin lactobionate was used by Yu et al. as CS for the NACE chiral separation of nine model basic molecules (amlodipine, atenolol, bisoprolol, esmolol, labetalol, metoprolol, nefopam, propranolol, ritodrine). The influence of CS concentration, BGE pH and composition, organic modifier type and concentration, and applied voltage on the enantio-resolution and migration times was studied. The best results were obtained when using 12.5 mM sodium borate BGE at pH 7.3–7.5 in a mixture with 50% methanol and 60 mM clarithromycin as CS. Another eight basic chiral drugs (chlorphenamine, chloroquine, citalopram, duloxetine, landiolol, sertraline, sotalol, trimetazidine) were tested but no chiral resolution was obtained. Utilizing Statistical Product and Service Solutions, a comparison of the influences of the analyzed parameters on enantio-separation was investigated using multivariate analysis of variance. Based on statistical results, CS concentration and BGE pH were the most important parameters that influence enantio-separation [58].

Lebedeva et al. used clarithromycin as CS in NACE for the chiral separation of 11 chiral drugs (amines and amino alcohols: alprenolol, atenolol, clenbuterol, fenoterol, labetalol, methoxyphenamine, metoprolol, pindolol, propranolol, sotalol, and synephrine). To optimize separation the influence of CS concentration, BGE pH, applied voltage, and the capillary temperature were studied. The best results were achieved in a methanolic solution of 100 mM citric acid, 10 mM sodium hydroxide, 240–300 mM boric acid, and 60–75 mM clarithromycin as CS. The method was applied for the quantification of metoprolol and propranolol enantiomers in pharmaceutical preparations [59].

**Figure 8:**
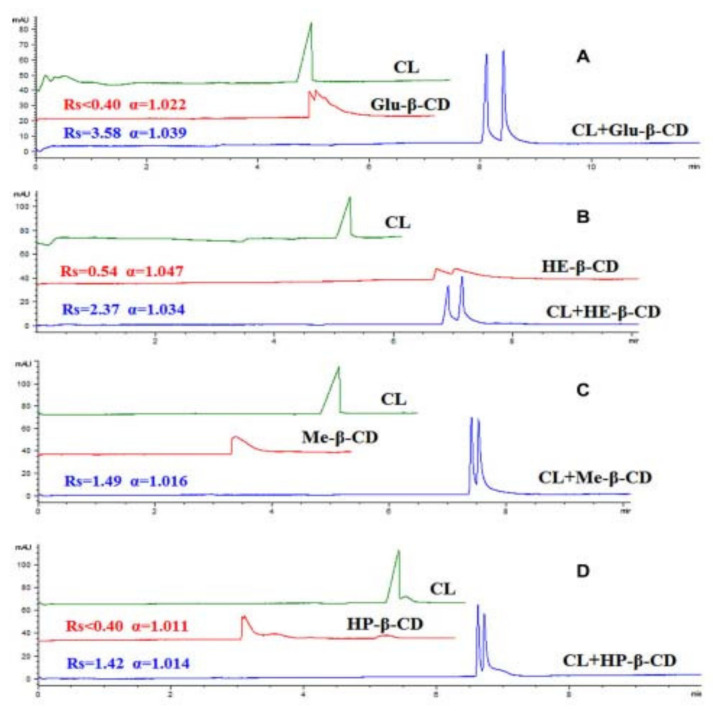
Enatio-separation of nefopam in single and dual CS systems (CL—clarithromycin, Glu-β-CD—glucose-β-CD, HE-β-CD—hydroxyethyl-β-CD, Me-β-CD—methyl-β-cyclodextrin, HP-β-CD—hydroxypropyl-β-CD) (CL concentration 50 mM, Glu-β-CD concentration 40 mM, HE-β-CD concentration 20 mM, Me-β-CD concentration 20 mM, HP-β-CD concentration 15 mM). (**A**)CL + Glu-β-CD; (**B**) CL + HE-β-CD; (**C**) CL + Me-β-CD; (**D**) CL + HP-β-CD. The figure is reproduced from Yu et al. [60] with permission from Wiley.

Yu et al. applied clarithromycin lactobionate in dual CS systems in combination with 4 neutral derivatized CDs (glucose-β-CD, hydroxyethyl-β-CD, hydroxypropyl-β-CD, methyl-β-CD) for the CE enantio-separation of nefopam. Nefopam was a substance that was only partially resolved in their previous study when clarithromycin lactobionate was used as a single CS [55]. The effects of CS concentration and BGE pH on enantio-resolution were studied. The use of all four dual CS systems resulted in baseline enantio-resolution (Figure 8). The separation of another six chiral drugs (atenolol, bisoprolol, esmolol, metoprolol, propranolol, ritodrine) was also evaluated with the four CS dual systems; synergistic effects were detected in all four CS systems. [60].

***Azithromycin*** is a semisynthetic macrolide containing a 15-membered macrocyclic lactone ring; derived from erythromycin, however, it differs from erythromycin as it contains a methyl-substituted nitrogen atom in the lactone ring. It has two sugar moieties, four hydroxyl groups, a methoxy group, and two tertiary amino groups. It is insoluble in water but quite soluble in alcohols, with low viscosity in these solvents and moderate UV absorption due to the lack of aromatic rings in the structure [18,52].

Azithromycin was used by Kumar and Park as CS in NACE for the enantio-separation of six chiral substances (carvedilol, cetirizine, citalopram, darifenacin, sertraline, tryptophan). As BGE, a polar organic mixture of acetonitrile, methanol, acetic acid, and triethylamine was used for enantio-separation. The effects of CS concentration, acetonitrile/methanol ratio, applied voltage, and capillary temperature on enantio-separation were examined. The best results were obtained using a BGE composed of acetonitrile/methanol/acetic acid/triethylamine (80:20:0.1:0.1%, *v/v/v/v*) and 4–6% (*w/v*) azithromycin as CS [61].

Lebedeva et al. used azithromycin as CS for the NACE enantio-separation of eight model basic derivatives (chlorpheniramine, doxylamine, hydroxyzine, isoproterenol, methoxyphenamine, synephrine, terbutaline, tetrahydrozoline). The effects of BGE composition, CS concentration, organic solvent type, capillary temperature, and applied voltage on the enantio-separation were studied. Enantio-separation was achieved only for two of the studied analytes, methoxyphenamine and tetrahydrozoline. The best results were obtained when using a BGE composed of 30 mM boric acid, 75 mM tributylamine, and 90 mM azithromycin as CS in methanol. LOD values for tetrahydrozoline were 5 µg/mL for both of the enantiomers. The method was applied to determine tetrahydrozoline in eye drops [62].

***Boromycin*** is a macrolide containing a stereogenic borate moiety. It is a macrodiolide Böeseken complex (J. Böeseken studied boric acid complexes with polyols containing a *d*-valine ester). It contains 16 stereogenic centers and has two hydroxyl groups, each attached to a stereogenic center. It is insoluble in water and soluble in polar organic solvents [63].

Maier et al. tested boromycin as CS in NACE for the chiral separation of six primary amines (2-amino-1-phenylethanol, *p*-hydroxynorephedrine, α-methylbenzylamine, norepinephrine, octopamine, and tryptophanol). The influence of CS concentration, BGE composition and concentration, and organic solvents type on enantio-resolution was established. The best results were obtained when using a BGE composed of 75 mM Tris and 50 mM boric acid in methanol, at pH 9.0 and 20 mM boromycin as CS. With the exception of tryptophanol, which was only partially resolved, practically all of the analytes were baseline separated. Tryptophanol’s low enantio-resolution was interpreted as a result of the stereogenic center’s increased distance from the aromatic moiety when compared with the structure of the rest of the amines [64].

***Gamithromycin*** is a second-generation macrolide used in veterinary medicine composed of a 15-ring structure with two sugar moieties. It has five hydroxyl groups, two tertiary amino groups, a methoxy group, and a propyl group associated with the N atom at the 7α position [65].

Ren et al. tested gamithromycin, as a new CS in NACE for the enantio-separation of nine amines (1-aminoindan, amlodipine, ethylamine, α-methylbenzylamine, 1-(4-methoxyphenyl) ethylamine, mexiletine, octapamine, pimaquine, and 1,2,3,4-tetrahydro-1-naphthylamine). The influence of BGE composition, CS concentration, type, and concentration of non-aqueous solvents, and applied voltage on the enantio-resolution was investigated. *N*-methylformamide was successfully used as a non-aqueous solvent, alone or in combination with methanol. The best results were obtained when using 100 mM Tris, 125 mM boric acid, and 80 mM CS in a mixture of methanol: *N*-methylformamide (25:75). The separation mechanism was also discussed, and it was concluded that enantio-recognition is based on the formation of hydrogen bonds between the CS hydroxyl group and the primary amine of the enantiomers. It is noticeable that gamithromycin exhibited remarkable enantio-selectivity towards amlodipine (resolution over 15) [65].

### 2.4. Lincosamides

Lincosamides are a small class of antibiotics that have amino acid and sugar moieties in their chemical composition. Lincomycin, the first native antibiotic of this class isolated from *Streptomyces lincolnensis*, can be considered structurally as a 4-propyl-proline derivative; the carboxyl group from proline acylates the amine group of an aminose [66].

**Figure 9:**
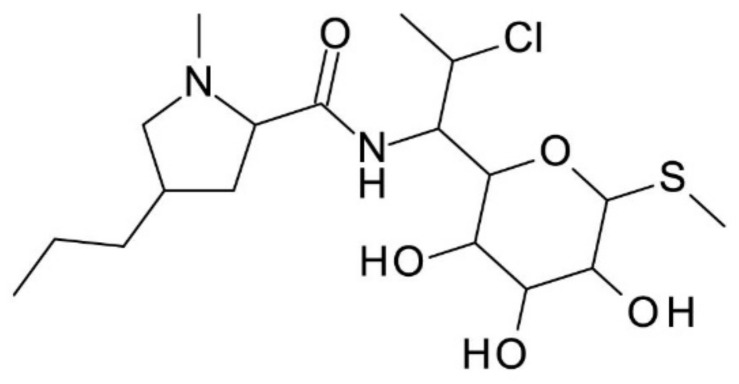
Chemical structure of Clindamycin.

**Clindamycin**, a semisynthetic derivative of lincomycin, a natural antibiotic produced by *Streptomyces lincolnensis*, is the only member of this family that has been used as a CS in CE. Clindamycin chemical structure is presented in Figure 9. In comparison to other macrocyclic antibiotics, clindamycin phosphate has a high solubility, as well as a low viscosity in water, and a low UV absorption due to the lack of aromatic rings in its structure [66].

Chen et al. used clindamycin phosphate as CS for the CE separation of eight basic chiral model drugs (atenolol, chlorphenamine, citalopram, metoprolol, nefopam, propranolol, tryptophan, and tryptophan methyl ester). The chiral separation of some acidic drugs was also investigated, without positive results. The influence of CS concentration, BGE pH, organic modifier, capillary temperature, and applied voltage on both migration time and enantio-separation was studied. The best results were obtained using a 40 mM sodium tetraborate BGE at neutral or weak basic pH (7.0–7.6), and a CS concentration of 60 to 80 mM. The organic modifier concentration also influenced the resolution and migration times of analytes, as well as the solubility of the CS; methanol and ethanol, among several organic solvents utilized, allowing the higher resolution. Furthermore, the effects of the analyzed parameters were studied using Statistical Product and Service Solutions [67].

**Figure 10:**
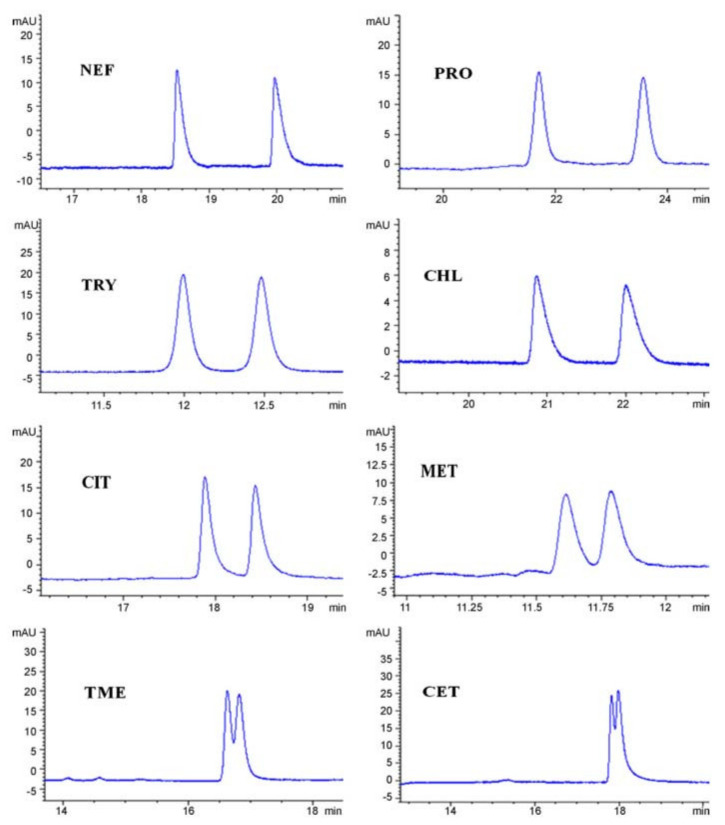
Enantio-separation of model basic drugs by MEKC using clindamycin phosphate as CS (Nefopam—NEF, PRO—propranolol, TRY—tryptophan, CHL—chlorphenamine, CIT—citalopram, MET—metoprolol, TME—tryptophan methyl ester, CET—cetirizine) (CS concentration 60 mM (NEF, PRO, CHL, TME, and CET) and 80 mM (TRY, CIT, MET). The figure is reproduced from Chen et al. [68] with permission from Elsevier.

The same research group used clindamycin phosphate as CS in micellar electrokinetic chromatography (MEKC) for the enantio-separation of model basic drugs (cetirizine, chlorphenamine, citalopram, metoprolol, nefopam, tryptophan, tryptophan methyl ester). In MEKC, an anionic surfactant is added to the BGE, generating a “pseudostationary” phase; differential partitioning of analytes between the “pseudostationary” phase and the bulk aqueous phase generating separation. Different types of anionic surfactants, organic additives, and BGE were tested, the best results were achieved using sodium dodecyl sulfate (SDS) as the surfactant, 2-propanol as an organic additive, and phosphate BGE. The influence of BGE pH and concentration, CS concentration, SDS concentration, 2-propanol concentration, and applied voltage on both migration times and enantio-resolution was examined. Satisfactory enantio-separations of the studied analytes were achieved, when using a 40 mM phosphate BGE at a pH range of 7.2–8 containing 40 mM SDS, 25% 2-propanol, and 60 mM or 80 mM clindamycin phosphate as CS (Figure 10) [68].

Wu et al. synthetized and used clindamycin succinate as a new CS in CE. The CS was tested on six basic model drugs (chlorphenamine, metoprolol, ofloxacin, propranolol, sotalol, tryptophan). Clindamycin succinate has better solubility in water or organic solvent than clindamycin phosphate, which has been used before for chiral separation of several basic drugs [67,68]. The effects of BGE pH, CS concentration, organic modifier, capillary temperature, and applied voltage on the enantio-separation were investigated; it was observed that enantio-resolution was strongly influenced by BGE pH and CS concentration. Good results were obtained when using a 50 mM Tris BGE at pH 4.0 containing 10% methanol and 50 mM clindamycin succinate as CS [69].

Wu et al. used a copper (II)-clindamycin CS for the enantio-separation of eight model basic drugs (atenolol, bisoprolol, epinephrine, esmolol, metoprolol, propranolol, sotalol, and tropicamide). The effects of the type of metal ion, the ratio of clindamycin and Cu(II), BGE pH, CS concentration, capillary temperature, and applied voltage on the enantio-separation were studied. The best results were obtained when using a BGE composed of 20 mM clindamycin/10 mM Cu^2+^ at pH 9.06. The high adsorption of the complex to the capillary inner surface is the fundamental drawback of this chiral separation method [70].

Ma et al. synthesized and used an ionic liquid (IL) CS based on tetramethylammonium clindamycin phosphate for CE enantio-separation of eight racemic analytes (chloroquine, chlorpheniramine, citalopram, hydroxychloroquine, nefopam, propranolol, tryptophan, tryptophanol). The influence of IL concentration, BGE pH, organic modifier proportion, and applied voltage on the enantio-separation was studied. The results were compared with the ones obtained with another IL CS, based on clindamycin phosphate, to investigate the influence of cation structure on the enantio-separation. Compared to native clindamycin phosphate, the results demonstrated that the tetramethylammonium clindamycin phosphate IL was superior as CS. In addition, molecular modeling was used to investigate the chiral recognition process of the IL CS, which indicated that the associated form of the IL has an important role in the enantio-separation. Moreover, the enantiomeric migration order could be predicted based on the calculated binding free energies of the enantiomers. This was the first time that an antibiotic-based IL CS has been used as the single CS in CE [71].

Xu et al. synthetized an IL CS containing cholinium-clindamycin phosphate and employed it as a sole CS in CE. The CS was tested on five model drugs (chlorpheniramine, citalopram, nefopam, propranolol, tryptophan). The influence of BGE pH, CS concentration, organic modifier, and applied voltage on the enantio-separation was verified. The IL selector demonstrated better enantio-separation capabilities and enhanced peak shapes under optimized conditions compared to clindamycin phosphate. The best results were obtained when using a 40 mM Tris BGE at pH 8.0 containing 20–40% methanol and 60 mM cholinium-clindamycin phosphate as CS. Molecular docking was employed in elucidating the chiral recognition process. Both the calculated free binding energies and the identified enantiomer-CS interactions indicated greater enantiomer affinities and enantioselectivity in the case of the IL CS in comparison to clindamycin. The computing results were in accordance with the experimental results [72].

### 2.5. Aminoglycosides

Aminoglycosides are heterozidic structure antibiotics, composed of an aglycon (genine) to which a variable number of dibasic aminocyclitoles (usually 2-deoxystreptamine) are glycosidically bound. Aminoglycosides show low UV absorbance due to the lack of aromatic rings in their structures [18,73].

Nishi et al. used diethylaminoethyl dextran hydrochloride (a polymer-type polysaccharide) and three different aminoglycoside antibiotics: fradiomycin sulfate, kanamycin sulfate, and streptomycin sulfate, as CS in CE for the enantio-separation of model acidic compounds (binaphthyl derivatives and synthetic intermediates of diltiazem analogues). A coated capillary was used to prevent the cationic CS from adhering to the inner surface of the capillary. The use of methanol helped to improve peak form and chiral resolution [73].

Zhang et al. studied the potential of streptomycin as CS in CE using as model molecules five phenyl-containing acidic drugs (adrenaline, dipivefrin, ibuprofen, isoprenaline, mandelic acid). The influence of CS concentration, BGE concentration and pH, capillary temperature, and applied voltage on the enantio-resolution was studied. The five substances were baseline separated in less than 10 min, on an uncoated silica capillary using a 20 mM phosphate BGE at pH 6.0 and 80 mM CS with UV detection at 214 nm. Streptomycin as CS presents a few advantages related to its good water solubility (it is used as a sulphate salt), and relatively low UV absorbance [74].

### 2.6. Other Antibiotics

***Benzylpenicillin*** (Penicillin G) is a natural β-lactam antibiotic. It has three chiral centers, a carboxylic group, and two amide groups. Low stability in acidic and basic environments represents one of the drawbacks of benzylpenicillin [75].

Benzylpenicillin potassium salt was tested as ion-pair CS in CE by Dixit and Park for the chiral separation of five basic drugs (citalopram, darifenacin, metoprolol, propranolol, and sertraline). The effects of BGE composition, capillary temperature, and applied voltage on the enantio-separation were evaluated. The addition of methanol in the BGE was essential to obtain enantio-resolution. The best results were obtained when using a BGE composed of water:methanol (90:10, *v/v*) and 10.7 or 16.1 mM benzylpenicillin as CS. The analytes were measured using indirect UV spectrophotometric detection, due to the strong UV absorption of benzylpenicillin. The stereoselective formation of diastereomeric ion-pairs between the negatively charged carboxylate group of the CS and the protonated amino group of the basic drugs was credited for benzylpenicillin enantio-separation ability [75].

***Doxycycline*** is a synthetic oxytetracycline derivative. It has six chiral centers, five hydroxyl groups, two carbonyl groups, and an amide group in its structure [76].

Jang et al. evaluated doxycycline as CS in NACE for the enantio-separation of 10 acidic model substances (carprofen, flurbiprofen, ibuprofen, ketoprofen, mandelic acid, suprofen, tropic acid, warfarin, and two amino acids: *N*-(3,5-dinitrobenzoyl)-leucine, *N*-(3,5-dinitrobenzoyl)-phenylglycine). The effects of CS concentration, BGE composition (containing acetonitrile/acetic acid/methanol/triethylamine), organic solvents (acetonitrile/methanol ratio, acetic acid, and triethylamine concentration), and applied voltage on the enantio-separation were studied. The best results were obtained when using a BGE composed of acetonitrile:methanol (50:50), 214 mM acetic acid, 63 mM triethylamine, and 38 mM doxycycline as CS. Because of doxycycline UV light absorption, indirect detection was used; this mode of detection monitors a small difference between two relatively big absorbance signals, resulting in high baseline noises, which is problematic in quantitative studies [76].

***Fusidic acid*** is a steroid type of topic antibiotic isolated from the fungus *Fusidium coccineum*. It has a steroid-like nucleus, 10 stereogenic centers, 2 hydroxyl groups, an acetyloxy group, and a carboxyl group. It has a pKa of 5.35, a neutral or basic BGE would be preferable for its CE use, as the ionization of the carboxyl group might enable complexation between fusidic acid and enantiomers through electrostatic interactions. It is insoluble in water (its sodium salt is soluble in water) but soluble in organic solvents [77].

**Figure 11:**
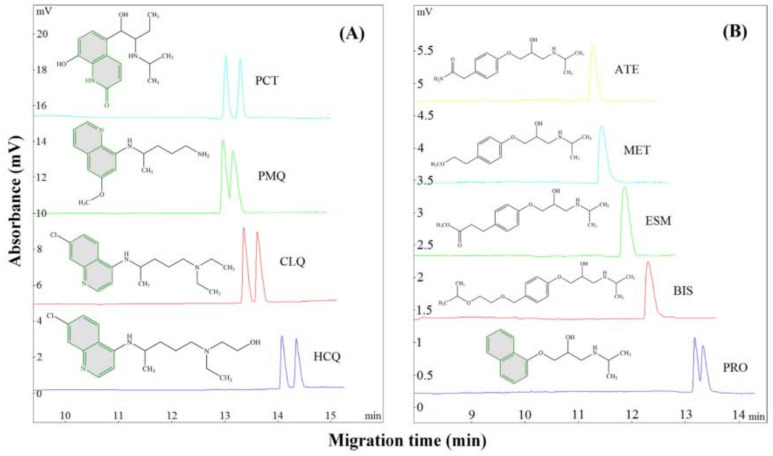
Enantioseparation of model drugs using fusidic acid as CS (PCT—procaterol, PMQ—primaquine, CLQ—chloroquine, HCQ—hydroxychloroquine, ATE—atenolol, MET—metoprolol, ESM—esmolol, BIS—bisoprolol, PRO—propranolol), (CS concentration 60 mM), (the rigid planar structure (if any) of model drugs is drawn in grey). (**A**) Enantioseparation of PCT, PMQ, ClQ and HCQ; (**B**) Enantioseparation of ATE, MET, ESM, BIS, PRO. The figure is reproduced from Zhang et al. [77] with permission from Elsevier.

Zhang et al. tested fusidic acid as a CS in CE, using as model molecules quinolone derivatives (chloroquine, hydroxychloroquine, and primaquine) and β-blockers (atenolol, bisoprolol, esmolol, metoprolol, and propranolol). The effects of BGE pH and concentration, CS concentration, organic modifier type and concentration on the enantio-resolution were studied. The best results were obtained with 50 mM phosphate buffer at pH 8.0 containing 60–70 mM CS and 50% methanol. Fusidic acid is particularly well suited to chiral analytes with a stiff planar structure (e.g., quinolone ring), according to a preliminary investigation of the separation process. One possible explanation is that the enantiomers with a rigid planar structure can induce a strong steric hindrance effect when interacting with the unique chair-boat-chair conformation fusidic acid. Among the five β-blockers, only propranolol contains a rigid planar structure (because of its naphthyloxy group); as expected, split peaks were obtained only in the case of propranolol using fusidic acid as CS (Figure 11) [77].

## 3. Discussion

Antibiotics are significant not just for their therapeutic use, but also for their ability to interact stereospecifically with chiral compounds. This stereospecific capacity is due to their structural characteristics and is based on the high number of stereogenic centers and different functional groups, which allows them to interact with chiral molecules in multiple ways (dipole–dipole interactions, electrostatic interactions, inclusion complexation, hydrogen bonding, π–π interactions) [78,79].

Macrocyclic antibiotics’ structural and chemical features are strongly linked to their chiral recognition abilities, but also impose certain limitations on their use as CSs in CE.

Several classes of antibiotics have demonstrated enantio-resolution power in the case of CE chiral separation: glycopeptides, macrolides, lincosamides, ansamycins, aminoglycosides, etc. [17,18,21].

Because of their great enantio-selectivity glycopeptide antibiotics (vancomycin, ristocetin, balhimycin, eremomycin, and others), are the most commonly used CS antibiotics in CE. The tendency of glycopeptide antibiotics to adsorb on the capillary wall and their high UV absorbance are the main drawbacks of their use. Coated capillaries (to avoid antibiotic adsorption) and partial filling or counter-current procedures (to avoid the presence of the CS in the detector) are commonly utilized to overcome these constraints [18,21].

Gasper et al. studied the impact of pH on the electrophoretic mobility of vancomycin, ristocetin A, and teicoplanin. To highlight their similarities and differences, both experimental and molecular modelling measurements were conducted. Vancomycin and ristocetin A proved to have very similar electrophoretic behavior in the studied pH range, as well as they showed close *pI* values, zero mobility, under the applied experimental conditions (7.2 and 7.5, respectively). Teicoplanin behaves differently, having a somewhat anionic nature even in an acidic environment. For analytes with significant binding constants to vancomycin and teicoplanin, adding an achiral surfactant (SDS) slightly above its critical micelle concentration reduces migration times, increases efficiency, and reverses the retention order. SDS, on the other hand, can either boost or reduce enantio-selectivity in a ristocetin A-based CE separation. The three glycopeptides have similar but not identical structures, resulting in comparable but not identical enantio-selectivities. This leads to the “principle of complementary separations,” which states that a partial resolution with one CS may be brought to baseline using another CS. Overall, the authors conclude that ristocetin A has the best potential for CE enantio-separations [22].

Ward et al. compared the enantio-recognition capacity of vancomycin and ristocetin A, and the potential of obtaining a synergistic effect when using a mixture of the two antibiotics as CS. Ristocetin A and vancomycin were chosen because of their similar *pI* values, consequently, were expected to migrate in the same direction at BGE pH values between 5 and 7. At the pH employed in this work, teicoplanin which has a *pI* value lower than that of ristocetin A and vancomycin would behave differently and migrate at a significantly different rate and direction. For drugs that exhibited enantio-selectivity to vancomycin, the mixed CS (containing 2 mM ristocetin A + 2 mM vancomycin) provided better enantio-separations than the use of 2- or 4 mm vancomycin as CS. On the other hand, the use of 4 mM ristocetin A, yielded better separations than those obtained with the mixed CS for compounds that showed stronger enantio-recognition with ristocetin A [80].

The glycopeptide antibiotics show strong absorption in the UV region, which may cause decreased sensitivity of detection. To avoid this counter-current mode in partial filling technique can be used. This approach maintains a BGE pH at which the CS and analyte have opposing charge forms and consequently move in opposite directions. In the partial filling technique prior to injection, the coated capillary is partially filled with a CS-containing BGE at the area between the detection window and the intake end, leaving the detector path free of the CS. The positively charged CS zone migrates to the cathode throughout the separation process, whereas the anionic analyte migrates to the anode; as a result, the CS could never reach the detection window, causing no analyte detection interference. Because the CS is not present in the detection cell, the partial filling technique improves analyte detection, and the counter current mode allows for high stereoselective interactions for the opposite migration of the CS and the analyte [81].

Macrolide antibiotics (azithromycin, clarithromycin, erythromycin) have gained popularity as CS in CE in the last 10 years. In contrast to macrocyclic antibiotics, they have lower UV absorption and are suitable candidates as CS in NACE because of their limited water solubility [18,21].

Macrocyclic antibiotics can be protonated at pH values lower than their *pI* due to the presence of amino groups in their structures, resulting in the adsorption of CSs on the capillary wall. The degree of adsorption is determined by the structural characteristics of antibiotics; it is larger when the antibiotic contains more amino groups and has a higher overall positive charge. Because of the peak widening caused by CS adsorption, the EOF may be reduced, resulting in prolonged migration times and a decrease in resolution.

Several experimental strategies for controlling the adsorption phenomenon have been documented in the literature, including optimizing the capillary washing procedure, changing the content and concentration of BGE, and using coated/modified capillaries [82].

In order to avoid adsorption different types of coatings have been used: covalently bonded polyacryl amide, poly(dimethylacrylamide), and chitosan coatings.

High temperatures, high applied voltages, and strongly acidic or basic pH conditions all compromise antibiotic stability. As a result, optimizing these variables is critical for effective and repeatable enantio-separations. Although deteriorated antibiotic solutions do not always result in the full loss of stereoselective characteristics and analyte interactions, noisier baselines, longer migration times, and lower enantiomeric resolutions can be observed.

Enantio-separation in CE is influenced by CS concentration, BGE pH and concentration, BGE organic additives, capillary temperature, and applied voltage.

The migration times and chiral resolution are affected by the concentration of CS. In general, as the concentration of CS increases, both analytical responses rise as well. Higher BGE viscosity, decreased EOF, and increased CS adsorption on the capillary wall all contribute to the longer migration time. The increased resolution is due to a higher degree of interaction between the CS and the analytes as a result of longer retention durations and increased complexation. However, peak widening and decreased resolutions were reported above the optimum concentration due to saturated complexation between CS and enantiomers. The concentration of CS of high UV absorbing selectors has also a significant impact on detection sensitivity.

In order to identify the best experimental conditions for successful enantio-separation, it is critical to choose the optimum BGE, in terms of pH, nature, and concentration. The BGE pH governs the charge and degree of ionization of both CSs and the analytes. To optimize electrostatic interactions, the BGE pH is kept at lower and higher values than the *pI* of the CS for enantio-separation of anionic and cationic analytes, respectively, to retain opposing charges on the CS and analytes. As the difference between migration times of the CS-enantiomer complex and the free enantiomer increases, the opposing charges on CS and analytes will help with the enantio-separation. Because of stability issues of antibiotics, a pH range of 4.0–9.0 is normally used when using them as CS.

Some critical parameters are affected by the BGE concentration: EOF, Joule heating, and CS adsorption on the capillary wall. Although concentrated BGE (50–100 mM) is useful in reducing CS adsorption on the capillary wall, the BGE high electroconductivity may produce EOF slowdown, baseline noise, and peak broadening due to increased Joule heating. Furthermore, high currents may have an unfavorable effect on CS stability.

Organic modifiers (acetonitrile, methanol, 2-propanol) can change or improve enantio-separation by affecting various parameters, such as BGE viscosity, analyte and CS solubility, CS adsorption on capillary walls, or inclusion complexation efficiency. An increase in the amount of organic modifier will decrease EOF magnitude and typically increases analyte retention times.

Temperature can alter binding stability and enantio-separation, as well as the migration time since enantio-discrimination is determined by the different binding abilities of two enantiomers to the CS. The weakening of the interaction between the analyte and the CS is attributable to a rise in capillary temperature, which produces a decrease in migration time and resolution. Because of the low stability of antibiotics, CE enantio-separations are usually carried out at temperatures between 15 and 25 °C.

The EOF magnitude, electrophoretic mobilities of analytes, and Joule heating are all affected by the applied voltage. A high applied voltage reduces analysis time and can improve peak sharpness. Increased Joule heating and unfavorable effects on CS stability, on the other hand, may be considered constraints of using higher voltages.

## 4. Conclusions

Macrocyclic antibiotics are now among the most extensively used CSs, being used in CE and HPLC for the enantio-separation of a large number of pharmaceuticals. Several experimental and review articles were published in the last 25 years regarding the use of certain macrocyclic antibiotics as CS in CE.

In the large majority of the articles published so far, the enantio-recognition capacity of macrocyclic antibiotics is just tested on a group of model chiral analytes (usually with the same structural characteristics). Very few studies have been published in which a macrocyclic antibiotic is used for the enantio-separation of a certain chiral drug, and even less for the enantio-separation of chiral analytes from complex matrices (biological matrices).

Although there are publications on the use of antibiotics in CE for chiral compound resolution, only a few applications are on real-life samples. Finding new CSs, optimizing separation methodologies, and building theory to describe the mechanism of enantio-separation have been the main goals of the studies published so far. Additionally, the use of antibiotics in dual CS systems remains an issue to be studied in the future.

CD and their derivatives remain by far the most efficient and frequently used CS in CE and macrocyclic antibiotics seem to be only an alternative CS; however, antibiotics have been shown to be sometimes effective in the separation of enantiomers of a variety of compounds, including pharmaceutical formulations, in a number of investigations.

## Abbreviations

BGE—background electrolyte; CD—cyclodextrin; CE—capillary electrophoresis; CEC—capillary electrochromatography; CE-ESI-MS—capillary electrophoresis–electrospray ionization-mass spectrometry; CS—chiral selector; CSP—chiral stationary phase; EOF—electroosmotic flow; FDA—Food and Drug Administration; GC—gas chromatography; HPLC—high performance liquid chromatography; LOD—limit of detection; LOQ—limit of quantification; IL—ionic liquid; MEKC—micellar electrokinetic chromatography; NACE—non aqueous capillary electrophoresis; NMR—nuclear magnetic resonance; pI—isoelectric point; SDS—sodium dodecyl sulfate; SFC—supercritical fluid chromatography.
